# Prevalence and Implications of Low Reticulocyte–Hemoglobin Levels among Extreme Preterm Neonates: A Single-Center Retrospective Study

**DOI:** 10.3390/nu14245343

**Published:** 2022-12-16

**Authors:** Jhanahan Sriranjan, Christine Kalata, Gerhard Fusch, Karen Thomas, Ipsita Goswami

**Affiliations:** 1 Department of Pediatrics, McMaster University, Hamilton, ON L8S 3L8, Canada; 2 Registered Dietitian, McMaster Children’s Hospital, Hamilton, ON L8N 3Z5, Canada

**Keywords:** prematurity, iron deficiency, anemia, reticulocyte hemoglobin, neurodevelopmental outcomes

## Abstract

This retrospective cohort study aims to determine the epidemiology of iron deficiency among extreme preterm neonates and the association of iron-deficient status during the NICU stay with neurodevelopmental outcomes at 18–24 months. Neonates ≤29 weeks gestational age (GA) born between June 2016 and December 2019, who received routine iron supplementation were enrolled. Iron deficiency was defined as reticulocyte–hemoglobin (Ret-Hb) levels ≤ 29 pg at 36 weeks corrected age. A subcohort of neonates completed standardized developmental assessment at 18–24 months corrected age. Significant neurodevelopmental impairment (sNDI) was defined as either Bayley Scales of Infant Development score < 70 or cerebral palsy or blindness or hearing aided. Among a cohort of 215 neonates [GA 25.8 (1.7) weeks, birthweight 885 (232) g], prevalence of iron deficiency was 55%, 21%, 26%, and 13%, in neonates <24 weeks, 24–25 + 6 weeks, 26–27 + 6 weeks, and ≥ 28 weeks GA, respectively. Male sex and receipt of corticosteroid therapy were associated with iron-deficiency. In the subcohort analysis (n = 69), there was no statistically significant association between Ret-Hb levels at 36 weeks corrected age and the risk of sNDI [OR 0.99 (95% CI 0.85–1.2)]. Male infants and those who received postnatal corticosteroids are likely to have iron-limited erythropoiesis at corrected term despite routine iron-supplementation; however, low Ret-Hb levels during the neonatal period were not associated with significant neurological disability in early childhood.

## 1. Introduction

Premature delivery ceases placental iron accretion that generally occurs during the third trimester of pregnancy [1,2]. This, combined with increased metabolic demands for growth and phlebotomy losses in the postnatal period, place preterm infants at risk of having poor iron stores [2]. At the same time, the preterm brain is undergoing neuronal growth, synaptogenesis, and pruning leading to differentiation of brain pathways crucial for future social, emotional, language, and intellectual functioning [3]. The fetal-neonatal brain is susceptible to iron deprivation because iron is a necessary micronutrient for rapidly proliferating and differentiating tissues [1]. Iron deficiency during this critical period of brain development is associated with neurocognitive problems, with lasting impacts extending into late childhood [4,5]. There is a known hierarchy in iron availability, which prioritizes erythropoiesis over brain development, and hemoglobin levels may not be affected until the iron stores are severely depleted [6]. Iron supplementation compensates for physiological insufficiencies and improves hemoglobin levels; however, it is unclear whether iron supplementation has long-term benefits on neurodevelopmental outcomes [7]. Therefore, knowledge about the iron status of preterm infants and understanding about its implications on the functional outcome will aid optimal management.

Hemoglobin levels from circulating erythrocytes (average lifespan of 120 days) and mean corpuscular volume (MCV) are late markers of iron deficiency [8]. Reticulocytes persist in the circulation for only 1–2 days and are thus more representative of current iron supply to the bone marrow. Iron-limited erythropoiesis can be recognized by low reticulocyte hemoglobin content (Ret-Hb), which has some advantages over other traditional measures of biochemical iron deficiency such as low serum ferritin and high zinc protoporphyrin [9,10]. Ferritin is an acute phase reactant, which is often abnormally elevated due to systemic inflammatory response. Zinc protoporphyrin is a measure of iron incorporation into erythrocytes and therefore does not reflect acute iron availability [11]. On the contrary, Ret-Hb reflects the functional iron available for erythropoiesis during the preceding 3–4 days and is thus a real-time indicator of bone marrow iron status [12]. Ret-Hb levels of 29 pg have been reported as the optimal cut-off for diagnosis of iron deficiency in preterm infants <32 weeks gestational age (GA) when using ferritin and transferrin saturation as gold standard comparators [10,13]. Normative reference standards for term and late preterm infants have noted 30 pg as the 10th centile for Ret-Hb distribution at birth [9]. The effect of Ret-Hb levels below the cut-off during critical neonatal growth phases on long-term outcomes remains largely unknown.

This study aims to (i) evaluate the prevalence of iron deficiency among preterm infants ≤ 29 weeks GA, as defined by Ret-Hb levels ≤ 29 pg, at 36 weeks postmenstrual age (PMA), and compare the perinatal risk factors between preterm infants with and without iron-deficiency; and (ii) examine the association between Ret-Hb levels at 36 weeks PMA and significant neurodevelopmental impairment at 18–24 months corrected age. We hypothesized that iron deficiency is present in most extreme preterm infants despite routine iron supplementation, and there is a significant negative association between Ret-Hb levels at 36 weeks PMA and severity of neurodevelopmental impairment.

## 2. Materials and Methods

Study design: We conducted a retrospective cohort study of extreme preterm neonates admitted to McMaster Children’s Hospital Level III Neonatal intensive care unit (NICU) between 1 June 2016 and 31 December 2019. For the primary aim of the study, we included a convenience sample of all consecutive eligible neonates admitted to the NICU during the study period. We performed a subcohort analysis of neonates who were admitted to the NICU between 1 June 2016 and 31 December 2017 and had completed their standardized developmental assessment at 18–24 months at the McMaster Developmental follow-up clinic by 31 December 2019 towards the secondary aim. The study was approved by the Hamilton Integrated Research Ethics Board (REB#13003). Clinical data were retrieved from archived medical health records of neonatal admission and developmental follow-up.

Study population: All consecutive inborn and outborn neonates born at or below 29 weeks GA were included in the study. Exclusion criteria were major chromosomal anomalies, congenital disorders, and discharge or death before 36 weeks PMA. As a unit policy, all preterm infants less than 2000 g birth weight are started on total parenteral nutrition on day 1 of life along with gavage feeds with either mom’s own milk or donor human milk. Feeds are increased according to weight-based criteria as long as there are no signs of feed intolerance. When tolerating 80 mL/kg/day, human milk fortification is added to feeds. All preterm infants less than 32 weeks GA were started on early standardized iron supplementation to compensate for physiological insufficiencies on day of life 14. Iron supplementation is started with a target to supplement with total of 3–4 mg/kg/day (birth weight <1 kg) or 2–3 mg/kg/day (birth weight >1 kg) to a maximum of 15 mg elemental iron from a combination ferrous sulfate supplements and human milk feeds with human milk fortifier. Dose of ferrous sulphate supplementation is revised from time to time according to infants’ weight gain, hemoglobin levels, ferritin level at 6–8 weeks. If ferritin levels are less than 80 mg/L, iron supplementation is targeted at 4–5 mg/kg/day. After recent blood transfusion ferrous sulphate supplementation is held for 7 days post-transfusion. McMaster Children’s Hospital is a tertiary care hospital, and neonates would be considered for discharge prior to 36 weeks PMA to surrounding Level 2 neonatal units, if clinically stable and either off respiratory support or on non-invasive CPAP less than/equal to 6 mmHg.

Hematological measurements: All preterm infants <32 weeks GA, if still admitted at 36 weeks PMA, have a routine complete blood count along with reticulocyte profiling carried out to optimize iron supplementation at 36 weeks PMA. Reticulocyte indices and reticulocyte counts are measured using Sysmex XN-10 hematology analyzer (Sysmex Corporation, Kobe, Japan). The automated analyzer is based on fluorescent flow cytometry using a semiconductor laser and hydrodynamic focusing in a dedicated reticulocyte channel. 

Outcome assessment: Primary outcome was defined as iron-deficient status with a Ret-Hb level ≤29 pg at 36 weeks PMA [10,13]. Given that Ret-Hb reflects iron availability for erythropoiesis in the last 2–3 days, we excluded datapoints from the final analysis if an erythrocyte transfusion was given to the neonates within 72 h prior to the measurement of Ret-Hb. If a Ret-Hb level was not carried out on the exact date that the neonate was 36 weeks PMA, we considered any Ret-Hb level measured between 35 and 37 weeks PMA for the analysis. Secondary outcomes included (i) Ret-Hb level at 4 weeks postnatal age; (ii) Hb level, Reticulocyte count, Mean corpuscular volume (MCV) at 4 weeks postnatal age, 36 weeks PMA and at discharge. For the subcohort analysis, a composite outcome was ascertained as significant neurodevelopmental impairment (sNDI), if any one of the following was present: (i) Bayley Scales of Infant Development Version 3 (BSID-III) score <70 in any domain, (ii) cerebral palsy with Gross Motor Function Classification system ≥3, (iii) bilateral visual impairment or (iv) hearing aided at 18–24 months corrected age. The BSID-III is a clinical evaluation of cognition, language, and motor skills in young children that has been validated to identify developmental delay (mean ± SD 100 ± 15) [14]. Secondary outcomes for the sub-cohort analysis assessed at 18–24 months included BSID III sub-scores in cognitive, language, and motor domain.

Variables: We collected patient demographics including (i) Perinatal data: birth-weight, GA, antenatal steroids (administration of two doses of corticosteroids during the concurrent pregnancy), clinical chorioamnionitis (maternal fever >38.4 °C at any time during labor, uterine tenderness during labor, leukocytosis of >15,000/mm^3^), Apgar score at 5 min, cord arterial blood pH or the first postnatal gas pH, severity of illness were collected using the Score for Neonatal Acute Physiology with Perinatal Extension version II (SNAPPE-II) score [15]; (ii) Comorbidities of preterm infants within 36 weeks PMA: Hemodynamically significant patent ductus arteriosus (PDA) requiring treatment (medical or surgical), Grade III-IV intraventricular hemorrhage (IVH) grading according to Papile’s classification [16], culture-proven sepsis (determined at least in a single positive blood culture), retinopathy of prematurity requiring Avastin or laser therapy, Stage II-III necrotizing enterocolitis (NEC) according to modified Bell’s criteria [17] and moderate to severe bronchopulmonary dysplasia (BPD) was defined based on the National Institute of Health consensus definition (2001) [18].

Statistical analysis: Descriptive statistics included frequencies and proportions (categorical variables) or mean and standard deviation (continuous variables). The cohort was stratified according to birth GA, and the proportion of infants who had Ret-Hb ≤ 29 pg at 36 weeks PMA was reported for each stratum. Perinatal characteristics between the two groups of neonates with and without Ret-Hb ≤29 pg at 36 weeks PMA were compared by Chi-square test or Student’s *t*-test as appropriate. A subcohort analysis was conducted to study the association between Ret-Hb level at 36 weeks PMA and sNDI by univariate logistic regression, and odds ratio (OR) and 95% confidence interval (CI) was reported. Spearman correlation analyses were performed to assess the correlation between the BSID subscore at follow-up in three individual domains (language, cognitive, and motor) and Ret-Hb levels to investigate the association between these two continuous variables. Statistical analysis was performed using GraphPad Prism Software Version 9.4.1.

## 3. Results

In total, 362 eligible neonates were admitted to the NICU during the study period. Among them, 217 neonates had outcome data available at 36 weeks PMA, and thus were included in the study. Two patients had received erythrocyte transfusion within 72 h of the measured Ret-Hb level and were excluded from the analysis. The reasons for outcome data being unavailable were either discharge to a Level II hospital prior to 35 weeks PMA (n = 91) or no complete blood count with reticulocyte profile recorded within 35–37 weeks PMA (n = 54). The neonates excluded from the study had higher birth GA and birth weight and were discharged earlier from the Level III neonatal unit to the Level II neonatal unit than the neonates included in the final cohort (Supplementary Table S1). For the subcohort analysis, among 85 eligible neonates who had Ret-Hb levels available at 36 weeks PMA, 69 infants completed standardized neurodevelopmental assessment at 18–24 months, and 16/85 (18%) were lost to follow-up.

### 3.1. Clinical Characteristics

The final cohort of 215 neonates had a mean (SD) birth GA of 25.8 (1.7) weeks, birth weight of 885 (232) g, SNAPPE score of 28 (19), and discharge GA of 41.8 (7) weeks. The median (IQR) number of erythrocyte transfusions received by the cohort during NICU stay was 3 (1–5). Among them, 47 (22%) had a Ret-Hb level of ≤29 pg at 36 weeks PMA. One hundred twenty-four infants had a complete blood count completed within a week prior to discharge, and 25/124 (20%) had a Ret-Hb level ≤29 pg at discharge. Table 1 compares the baseline characteristics of the neonates who met and did not meet the criteria for iron deficiency at 36 weeks PMA. At 36 weeks PMA, 22 (10%) neonates had severe BPD [treated with oxygen >21% for 28 days and needing oxygen >30% and/or invasive/noninvasive positive pressure at 36 weeks PMA], 40 (19%) neonates had moderate BPD [treated with oxygen >21% for 28 days and needing oxygen <30% at 36 weeks PMA], 114 (53%) neonates had mild BPD [treated with oxygen >21% for 28 days but breathing in room air at 36 week PMA] and 39 (18%) neonates had no BPD [in room air before 28 day of life].

A significantly higher number of neonates in the iron-deficient group were male infants and had received postnatal corticosteroids. Although they did not reach statistical significance, neonatal morbidities such as sepsis, necrotizing enterocolitis, bronchopulmonary dysplasia, and hemodynamically significant PDA tend to be higher in the iron-deficient group.

Neonates included in the subcohort analysis (n = 85) had a mean GA of 25.7 (1.8) weeks, birth weight of 838 (223) g, SNAP-II score of 31 (20) and discharge GA of 41.1 (5.2) weeks. Among them, 29/85 (34%) were male, 27/85 (31%) had delayed cord clamping, 7/85 (8%) were outborn, 40/85 (47%) received antenatal steroids, 11/85 (13%) had clinical chorioamnionitis. The incidence of neonatal morbidities among the subcohort was as follows: hemodynamically significant PDA 32/85 (38%), moderate-severe BPD 18/85 (21%), culture-proven sepsis 19/85 (22%), retinopathy of prematurity needing treatment 13/85 (15%) and stage II-III NEC 5/85 (6%).

### 3.2. Hematological Parameters

The prevalence of iron deficiency at 36 weeks PMA was 5/9 (55%) for neonates born at 23–24 weeks, 17/82 (21%) for neonates born at 25–26 weeks, 19/73 (26%) for neonates born at 26–27 weeks, and 6/46 (13%) for neonates born at 28–29 weeks. At 36 weeks PMA, hemoglobin level of neonates with Ret-Hb ≤29 pg was slightly lower than neonates with Ret-Hb level >29 pg, but the mean difference was not clinically significant. A comparison of other hematological parameters has been enumerated in Table 2.

**Figure 1:**
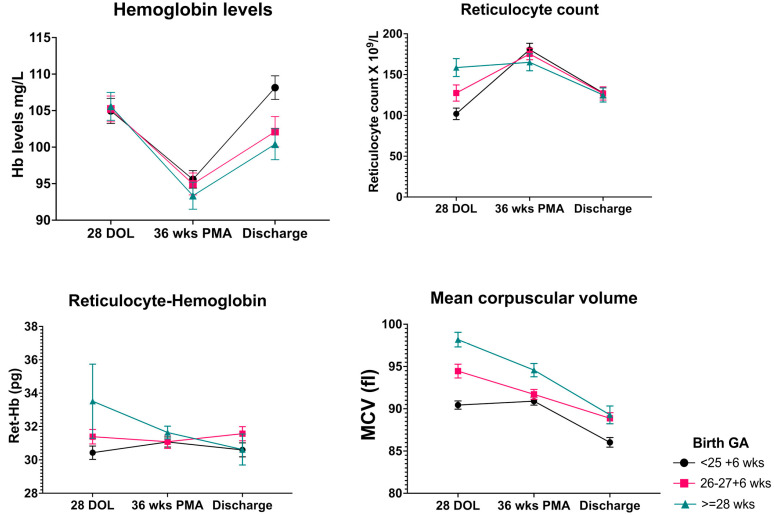
Longitudinal changes in hematological parameters during NICU admission in extreme preterm neonates. Mean and SEM of each hematologic variable specific to each age group is plotted to depict the natural history of changes over time. There is a tendency towards decreasing Hb levels and corresponding increase in reticulocyte counts between 28 days of life and 36 weeks PMA. On the contrary Ret-Hb levels remain unchanged during the NICU course, but MCV levels fall significantly with increasing postnatal age. Abbreviations: GA, Gestational Age; DOL, Days of Life; PMA, Postmenstrual Age; Hb, Hemoglobin; Ret-Hb, Reticulocyte Hemoglobin; MCV, Mean Corpuscular Volume.

**Figure 2:**
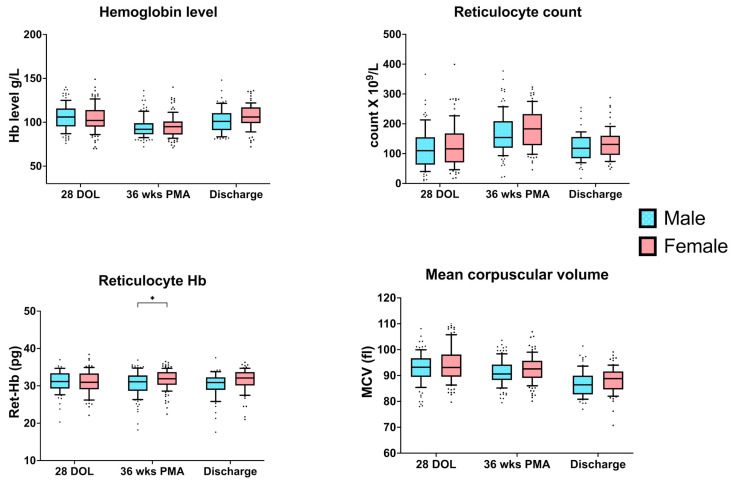
Sex differences in hematological parameters at three time points. Median (IQR) of each hematological variable specific to each sex is plotted. Unpaired Student’s *t*-test with False Discovery rate (FDR) approach for multiple comparison. * *p* value < 0.05. Although at all time points female infants tend to have higher values of hematological parameters compared to male infants, the mean difference in Ret-Hb levels at 36 weeks PMA reached statistical significance. Abbreviations: DOL, Days of Life; PMA, Postmenstrual Age; Hb, Hemoglobin; Ret-Hb, Reticulocyte Hemoglobin; MCV, Mean Corpuscular Volume.

Neonates, irrespective of their GA at birth, showed an initial decrease in mean hemoglobin levels from 4 weeks postnatal age to 36 weeks PMA, followed by an increase at the time of discharge from NICU. The changes in mean hemoglobin level were accompanied by a corresponding increase and subsequent decrease in reticulocyte count. Mean Ret-Hb levels remained mostly unchanged between the three time points of assessment. There was a significant effect of birth GA and postnatal age on MCV values. Mean MCV values at 4 weeks postnatal age tend to be lower in the lower GA group, and a further decrease in mean MCV values was noted at 36 weeks PMA and at discharge (Figure 1). There was a tendency towards female infants to have higher mean values of Hb, Ret-Hb, MCV, and Reticulocyte count than male infants at all time points but only mean difference in Ret-Hb levels at 36 weeks PMA reached statistical significance (Figure 2).

### 3.3. Neurodevelopmental Outcomes

**Figure 3:**
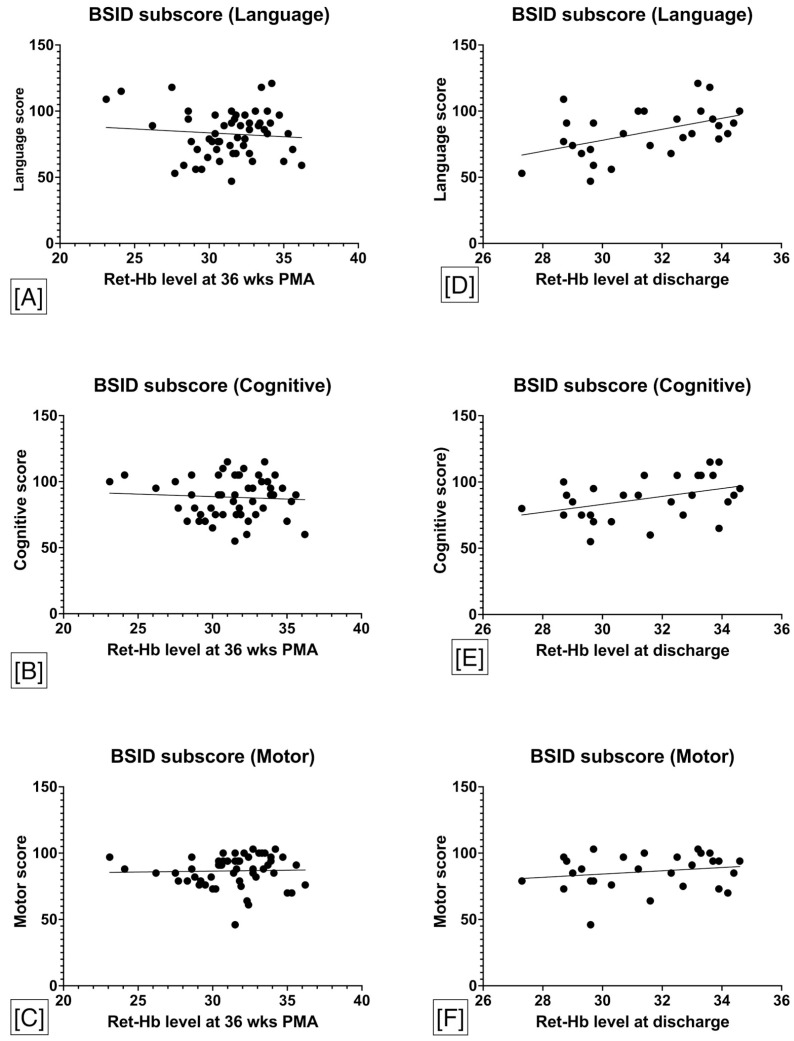
Association between Ret-Hb levels and BSID subscores in each domain at 18–24 months. There was no significant association between Ret-Hb levels at 4 weeks postnatal age and 36 weeks PMA with BSID-III subscore in cognitive (**B**), motor (**C**), and language (**A**) domains. However, Ret-Hb levels at discharge had a significant correlation with BSID-III subscores on language (**D**) domain and cognitive (**E**) domain, but not in motor (**F**) domain. Abbreviations: Ret-Hb, Reticulocyte hemoglobin; PMA, postmenstrual age; BSID, Bayley Score for infant development.

On subcohort analysis, 31/85 (36%) met the criteria for sNDI and 16/85 (18%) were lost to follow up. There was no significant difference in the incidence of sNDI between the neonates with Ret-Hb levels ≤29 pg and >29 pg at 36 weeks PMA [6/13 (46%) versus 25/56 (44%), *p* = 0.48]. On univariate logistic regression, there was no statistically significant association between Ret-Hb levels at 36 weeks PMA and the risk of sNDI [OR 0.99 (95% CI 0.85–1.2), *p* = 0.95]. There was no significant association between Ret-Hb levels at 4 weeks postnatal age and 36 weeks PMA with BSID-III subscore in cognitive, motor, and language domains (Figure 3). However, Ret-Hb levels at discharge had a significant correlation with BSID-III subscores on language domain [r = 0.43, *p* = 0.02] and cognitive domain [r = 0.38, *p* = 0.04], but not in motor domain [r = 0.13, *p* = 0.48].

## 4. Discussion

Our findings suggest that one-fourth of neonates born at 29 weeks GA or below and needing tertiary care support at or beyond 36 weeks PMA have Ret-Hb levels below the cut-off (29 pg), which may be indicative of inadequate iron availability for erythropoiesis. Although low Ret-Hb levels during the neonatal period was not associated with significant neurological disability in early childhood, we noted a positive correlation between Ret-Hb level at discharge and language/cognitive scores at 18–24 months suggesting that increasing iron stores with iron supplementation may have a positive influence on language and cognitive development in early childhood. 

Ret-Hb has the highest overall sensitivity and specificity compared with ferritin, transferrin saturation, and MCV for predicting the absence of bone marrow iron stores, provided MCV < 100 fL and in the absence of hemoglobinopathies [19]. Using a cut-off level of 29 pg, our findings indicate that iron availability for erythropoiesis may be adequate in majority (78%) of extreme preterm infants at 36 weeks PMA. Amin et al. used a cut-off value of 27 pg in a cohort of neonates <32 weeks GA and reported >79% of the cohort were iron repleted at 36 weeks PMA [20]. Compared to Amin et al., our cohort was relatively lower gestational age. Furthermore, the prevalence of iron deficiency at 36 weeks PMA was higher in lower birth gestational age groups, possibly explained by (1) iron endowment progressively increases with an increase in gestation during the last trimester; (2) higher GA infants are likely to have fewer phlebotomy losses; and (3) less likely to have other neonatal morbidities.

Neonates < 32 weeks’ gestation are at higher risk of abrupt postnatal decrease in hemoglobin levels along with inappropriately low reticulocyte count and circulating erythropoietin concentration for the degree of anemia [21]. Concurrent cardiorespiratory and infectious illnesses, the need for bloodwork, and developmentally regulated physiologic processes (i.e., decreased erythropoietin production, shorter lifespan of fetal erythrocytes) contribute to progressive anemia [22]. We assessed multiple hematological parameters at three different time points during the NICU course and observed—age-related changes in hemoglobin, reticulocyte count, and MCV values, but not Ret-Hb levels. MCV values were negatively correlated with birth GA at all time points, which has been previously reported in preterm neonates [9]. Previous studies have reported similar trends, i.e., initial decrease in hemoglobin levels reaching a nadir at 6 weeks postnatal age followed by an increase at 3 months, concurrent with reciprocal changes in reticulocyte count and steady decrease in MCV values which stabilizes at 6 months [23,24]. Our findings are in keeping with these trends, except for the fact that hemoglobin levels continue to drop beyond 4 weeks postnatal age up to 36 weeks PMA. This is not surprising, given our cohort had a much lower gestational age (mean 25 weeks) than previously reported studies (>28 weeks), and it is likely that the least mature infants are susceptible to more pronounced decline and a later nadir.

Age and gestational maturity related differences in blood parameters may reflect differences in erythropoietic activity. It has been previously reported that smaller preterm infants (<1500 g) have higher erythropoietic activity than bigger preterm infants (>1500 g) [24]. Immediately after birth, abrupt increase in oxygen delivery to the tissues results in decline in plasma erythropoietin and a marked decline in erythroid precursors in the bone marrow within the first postnatal week [25]. Iron demands for erythropoiesis are lowest during the early neonatal period due to cessation of erythropoiesis [20] but gradually increase over time, given the rapid growth rate in the first year of life, increasing the need for iron [24]. Our data show that despite changes in erythropoietic activity (reticulocyte count) over time, Ret-Hb levels were not significantly affected by postnatal age beyond 4 weeks of life. Similar observations were made by German et al., who reported that Ret-Hb levels were lowest around 14 days and returned to the baseline by 42 days; subsequently, there is minimal change over time until 120 days of life [11]. Two other studies reported that Ret-Hb levels are highest at birth, decreases thereafter but consistently flattens around a mean of 30.0 pg by the 3–4 months of life [9,26] The stagnancy of Ret-Hb levels may be attributed to routine iron supplementation with dose adjustments with increasing postnatal age and weight. Importantly, our data generate the hypothesis that assessment of the erythropoietic activity (reticulocyte count) and functional iron availability (Ret-Hb) may help to tailor therapeutic decisions to physiological needs. For instance, in an otherwise thriving neonate with low hemoglobin, (1) both high Ret-Hb and reticulocyte counts may indicate sufficient capacity to correct anemia without further intervention; (2) low levels of reticulocyte but high levels of Ret-Hb may indicate there is enough iron but ineffective erythropoiesis; (3) low Ret-Hb and high reticulocyte counts may indicate effective erythropoiesis, but not enough iron available for normal hemoglobinization.

Two clinical risk factors were associated with iron deficiency: exposure to postnatal corticosteroids and male sex. Exogenous corticosteroid therapy stimulates erythropoiesis [27] and hence may lead to relative iron deficiency if iron supplementation is not increased to meet the increasing demands. Gender differences in iron status have been reported previously. Boys have significantly lower Ret-Hb compared to girls at 4 months of age [28]. Choi et al. reported male infants had higher soluble transferrin receptor than females both in cord blood and at 4–6 months of age [29]. Similarly, at 4 months boys tend to have lower hemoglobin, MCV, ferritin, and higher zinc protoporphyrin and transferrin receptors than girls even after controlling for birth weight and postnatal weight gain [30]. Although unexplained, some speculations towards male infants’ propensity to iron deficiency are (1) higher erythropoietic activity; (2) lower iron stores at birth; (3) higher intestinal iron losses; (4) lower iron absorption; (5) higher rate of infections and neonatal morbidities which may affect iron status. 

Neonates who had lower Ret-Hb levels by the time of discharge tend to have lower cognitive and language score by 2 years of age, thus raising the possibility that iron-limited erythropoiesis at the bone marrow may be associated with poor development of specific brain regions involved in language development and cognitive skills. A significant part of preterm brain development occurs extrauterine, especially the structural development of the hippocampus [31,32,33]. It is also believed that the temporal and regional emergence of myelination in the auditory pathway occurs during 36–40 weeks GA [34]. During periods of iron limitation, iron is preferentially routed towards erythropoiesis at the expense of brain iron accretion [6] therefore, the effect of iron deficiency on brain development is perhaps independent of the primary effects of anemia. Our findings are concurrent with some other studies, including Tamura et al., who reported poorer performance in fine motor skills and language development at 5 years in late preterm and term infants born with a cord serum ferritin level <76 ng/mL [35]. Another study found that term infants with cord serum ferritin level ≤34 mg/L had poor auditory recognition memory and lower psychomotor scores at 1 year [36]. In utero latent iron deficiency is associated with abnormal auditory neural maturation in near term infants [34,37]. As infant’s reach closer to corrected term gestation erythropoietic activity increases, hence, increasing the iron demands coinciding with brain developmental process during phases of region-specific growth spurts and periods of vulnerability. This hypothesis will need to be tested prospectively in future. 

Our study had certain limitations. Firstly, given the retrospective study design, only infants who had a complete blood count at 35–37 weeks PMA could be included in the study. It is possible that the Ret-Hb levels were available only in infants who needed tertiary care support beyond 36 weeks PMA, thus representing a bias in our cohort towards sicker and less mature infants. However, we do note that most previously published reference ranges are from infants >28 weeks of gestation, and thus our data add to the literature by evaluating the most vulnerable infants. Secondly, only a small proportion of infants had neurological assessment completed within the study period. Given the small number of neurodevelopmental outcomes available, multivariate analysis was not feasible. Due to the multifactorial influence of GA, perinatal morbidities, brain injury and postnatal nurturing on neurodevelopmental outcome, a larger sample size would be needed to identify any independent effects of iron status on neurodevelopmental outcomes. Third, the association between BSIDIII subscore and Ret-Hb levels at discharge will need to be considered with caution, since the corrected GA at discharge was not constant for all infants. Finally, we were not able to collect data on maternal iron deficiency or cord blood iron status, which may be an important determinant of iron status in the neonatal period. Measuring serum ferritin was not a consistent unit practice during the study period, hence no other biochemical test of iron status was available for the cohort. Nevertheless, our study adds to the literature by highlighting the potential role of Ret-Hb in understanding dynamic aspects of iron metabolism and erythropoiesis in preterm infants. Our findings better define the natural history of Ret-Hb values in a preterm cohort most at risk of iron deficiency, yet are underrepresented in prior literature. To the best of our knowledge, the long-term functional outcomes of Ret-Hb levels in neonatal period has not been reported before. The effect of supplementation in the most vulnerable infants, particularly those born very preterm or with a birth weight < 1500 g, is yet to be elucidated [38]. Our results lay the foundation for future studies to determine the effect of iron supplementation, above and beyond the potential effect of other comorbidities, on neurodevelopmental outcome and, whether both sexes are equally susceptible to the functional consequences of iron deficiency.

## 5. Conclusions

In summary, most extreme preterm infants receiving elemental iron supplementation are iron replete for erythropoiesis by term corrected GA. However, male infants and those exposed to postnatal corticosteroid therapy are at risk of poor iron stores. Functional iron availability for erythropoiesis at discharge has a positive correlation with language and cognitive development in early childhood. Future longitudinal studies would be needed to gain insight into differential gender relationships between iron stores and iron availability, and the need for sex-specific cut-offs. Potential benefits of tailored iron supplementation will need confirmation in a well-designed trial.

## Figures and Tables

**Table 1:** Perinatal characteristics of neonates with and without iron limited erythropoiesis at 36 weeks corrected gestational age.

	Ret-Hb > 29	Ret-Hb ≤ 29	*p* Value
	N = 168	N = 47	
Gender (Male) n (%)	74 (44)	31 (65%)	0.008
Gestational age (weeks) mean (SD)	26 (1.6)	25.5 (1.6)	0.07
Birth Weight (g) mean (SD)	898 (237)	837 (207)	0.11
Apgar score at 5 min median (IQR)	7 (6–8)	7 (6–8)	0.99
Cord arterial pH or postnatal gas pH	7.28 (0.09)	7.29 (0.08)	0.53
Delayed Cord Clamping n (%)	75 (44)	22 (46)	0.81
SNAPPE-II score mean (SD)	26.7 (18)	31.3 (21)	0.15
No of transfusions median (IQR)	3 (1–5)	3 (1–5)	0.68
Gestation at discharge mean (SD)	41.6 (6.9)	42.5 (8.4)	0.45
Small for gestation n (%)	11 (6.5)	1 (2.1)	0.24
Vaginal delivery n (%)	67 (39)	16 (34)	0.46
Outborn n (%)	28 (16%)	9 (19%)	0.69
Antenatal Steroids n (%)	93 (55%)	20 (42%)	0.12
Chorioamnionitis n (%)	45 (28%)	14 (32%)	0.56
Sepsis n (%)	48 (28%)	16 (34%)	0.46
Necrotising Enterocolitis n (%)	12 (7%)	6 (13%)	0.21
Postnatal steroids n (%)	85 (50%)	33 (70%)	0.02
Laparotomy n (%)	15 (8%)	6 (12%)	0.78
Hemodynamically significant PDA n (%)	68 (39%)	23 (48%)	0.48
Mod-severe Bronchopulmonary Dysplasia n (%)	44 (30%)	18 (40%)	0.14
Retinopathy of Prematurity treatment n (%)	20 (12%)	7 (14%)	0.58
Mod-severe Intraventricular hemorrhage n (%)	24 (14%)	9 (19%)	0.41

Categorical variable Chi-square or Fisher-exact test, Continuous variable Student’s *t*-test. Abbreviations: PDA; Patent Ductus Arteriosus, SNAPPE-II; Score of Neonatal Acute Pathologies with Perinatal Extension version II.

**Table 2:** Comparing ancillary hematological parameters between neonates who meet and do not meet criteria for iron deficiency at 36 weeks corrected gestational age.

	Ret-Hb > 29 pg	Ret-Hb ≤ 29 pg	*p* Value
	N = 168	N = 47	
Hemoglobin at day 28 (g/L)	103 (96–115)	103 (92–116)	0.85
Hemoglobin at 36 weeks PMA (g/L)	94 (87–101)	89 (84–96)	0.04
Hemoglobin at discharge (g/L)	105 (93–114)	105 (96–117)	0.45
Reticulocyte count at day 28 (×10^9^/L)	116 (72–161)	130 (53–130)	0.06
Reticulocyte count at 36 weeks CGA (×10^9^/L)	163 (120–213)	182 (137–222)	0.23
Reticulocyte count at discharge (×10^9^/L)	129 (92–160)	106 (89–141)	0.36
Ret-Hb at day 28 (pg)	31.5 (29.4–33.4)	30.2 (27.3–33)	0.04
Ret-Hb at discharge (pg)	31.9 (30–33)	29.7 (27–31)	0.002
MCV at day 28 (fL)	93.4 (89–97)	91.9 (87–96)	0.05
MCV at 36 weeks PMA (fL)	91.9 (89–95)	90.2 (87–95)	0.07
MCV at discharge (fL)	88.7 (84–91)	84.7 (81–88)	0.001

Mann–Whitney U test (non-parametric variables), Student’s *t*-test (normal distribution variables). Abbreviations: PMA, Postmenstrual Age; MCV, Mean Corpuscular Volume; CGA, Corrected Gestational Age.

## Data Availability

Data are contained within the article or supplementary material.

## Supplementary Materials

The following supporting information can be downloaded at: https://www.mdpi.com/article/10.3390/nu14245343/s1, Table S1: Comparison of baseline characteristics between cohort of neonates included and excluded from the analysis.
