# Supplementary material for: Prevalence and Implications of Low Reticulocyte–Hemoglobin Levels among Extreme Preterm Neonates: A Single-Center Retrospective Study
Source: Nutrients. 2022 Dec 16;14(24):5343. doi: 10.3390/nu14245343 (PMC9788547; doi:10.3390/nu14245343)
Supplement: Supplementary file 1 [file nutrients-14-05343-s001.zip › nutrients-2049017-supplementary.pdf]

Table S1: Comparison of baseline characteristics between cohort of neonates included and excluded from the analysis

	Included Cohort	Excluded Cohort	p value
	N = 215	N = 147	
Gender (Male) n (%)	105 (49)	87(59)	p = 0.05
Gestational age(weeks) mean (SD)	25.8 (1.7)	26.6 (1.5)	p <0.0001
Birth Weight (g) mean (SD)	885 (232)	1019 (254)	p <0.0001
Delayed Cord Clamping n (%)	97 (45)	75(51)	p = 0.23
SNAPPE score<br>mean (SD)<br>median (IQR)	27.8 (19)<br><br>24 (14-41)	23.6(19)<br><br>20(9-34)	p = 0.05
Gestation at discharge (weeks)<br>Mean (SD)<br>median (IQR)	41.8 (7)<br><br>40 (37-45)	36.4 (7)<br><br>34 (32-37)	p <0.0001
Small for gestation n (%)	12(5.5)	6(4)	p=0.51
Vaginal delivery n (%)	83 (38)	64(43)	p=0.35
Outborn n (%)	37 (17)	21(14)	p=0.45
Antenatal Steroids n (%)	113(52)	85(57)	p=0.32
Histological Chorioamnionitis n (%)	59(29)	28(20)	p=0.06
Sepsis n (%)	64(30)	34(23)	p=0.16
Necrotising Enterocolitis n (%)	18 (8)	8(5)	p= 0.28
Hemodynamically significant PDA n (%)	91(42)	45(31)	p=0.07
Mod-severe Bronchopulmonary Dysplasia n (%)	66(31)	45(30)	p = 0.61
ROP needing treatment	27 (12)	9(6)	0.05

Categorical variable Chi-square or Fisher-exact test, Continuous variable Student-t-test.
